# Supplementary material for: Heat adaptation of phage T7 under an extended genetic code
Source: Virus Evol. 2021 Dec 1;7(2):veab100. doi: 10.1093/ve/veab100 (PMC8923235; doi:10.1093/ve/veab100)
Supplement: veab100_Supp [file veab100_supp.zip › Sequencing_Analysis.zip!/Sequencing_Analysis/A3/evidence/RA_43.html]

BRESEQ :: Evidence


| Read alignment evidence... | | | | | | | | | | | |
| --- | --- | --- | --- | --- | --- | --- | --- | --- | --- | --- | --- |
|  | seq id | position | | ref | new | freq | score (cons/poly) | reads | annotation | genes | product |
| \* | NC\_001604.1 | 30,576 | 0 | C | T | 51.9% | 56.2 / inf | 1629 | intergenic (–/–) | *–/–* | –/– |
| Reads supporting (aligned to +/- strand):  **ref** base C (379/399);  **new** base T (434/405);  **total** (818/811) | | | | | | | | | | | |
| Fisher's exact test for biased strand distribution *p*-value = 2.33e-01 | | | | | | | | | | | |
| Kolmogorov-Smirnov test that lower quality scores support variant *p*-value = 8.57e-01 | | | | | | | | | | | |

Only 100 of 1641 total aligned reads displayed.  

`AGCAACCCTTGGATAACCAATAAGCAACTGACCATGTATTCTCAAGGTGACTCCATTTACCTTATGGACACCACAGGTCAAGTCAGAGTCCGATACGACAAAGAGTTACTCTCGAAGGTCTGGAGTGAGAACCAGAAGAAACTCGAAGAGAAAGCTCGTGAGAAGGCTCTGGCTGATGTGAACAAGCGAGCACCTATAGTTGCCGCTACGAAGGCCCGTGAAGCTGCTGCTAAACGAGTCCGAGAGAAACGTAAACAGACTCCTAAGTTCATCTACGGACGTAAGGAGTAACTAAAGGCTACATAAGGAGGCCCTAAATGGATAAGTACGATAAGAACGTACCAAGTGATTATGATGGTCTGTTCCAAAAGGCTGCTGATGCCAACGGGGTCTCTTATGACCTTTTACGTAAAGTCGCTTGGACAGAATCACGATTTGTGCCTACAGCAAAATCTAAGACTGGACCATTAGGCATGATGCAATTTACCAAGGCAACCGCTAAGGCCCTCGGTCTGCGAGTTACCGATGGTCCAGACGACGACCGACTGAACCCTGAGTTAGCTATTAATGCTGCCGCTAAGCAACTTGCAGGTCTGGTA  >  NC_001604.1/30278‑30876`  

                                                                                                                                                                                                                                                                                                          `|`                                                                                                                                                                                                                                                                                                              
    `ataagagacagaCCAATAAGCAACTGACCATGTATTCTCAAGTTGACTCCATTTACCTTATGGACACCACAGGTCAAGTCAGAGTCCGATACGACAAAGAGTTACTCTCGAAGGTCTGGAGTGAGAACCAGAAGAAACTCGAAGAGAAAGCTCGTGAGAAGGCTCTGGCTGATGTGAACAAGCGAGCACCTATAGTTGCCGCTACGAAGGCCCGTGAAGCTGCTGCTAAACGAGTCCGAGAGAAACGTAAACAGACTCCTAAGTTCATCTACGGACGTAAGGAGTAACTAAAGGCTACATa                                                                                                                                                                                                                                                                                                        <  1:103791/290‑1 (MQ=255)`  
    `aCCCTTGGATAACCAATAATCAACTGACCATGTATTCTCAAGGTGACTCCATTTACCTTATGGACACCACAGGTCAAGTCAGAGTCCGTTACGACAAAGAGTTACTCTCGAAGGTCTGGAGTGAGAACCAGAAGAAACTCGAAGCGAAAGCTCGTGAGAAGGCTCTGGCTGATGTGAACAAGCGAGCACCTATAGTTGCCGCTACGAAGGCCTGTGAAGCTGCTGCTAAACGAGTCTGAGAGAAACGTAAACAGAATCCTAAGATCATCTACGGACGTACGGAGTAACTAAAGGTTaatta                                                                                                                                                                                                                                                                                                        >  1:301722/1‑297 (MQ=255)`  
     `cccTTGGATAACCAATAAGCAACTGACCTTGTATTCTCAAGGTGACTCCATTTACCTTATGGACACCACAGGTCAAGTCAGAGTCCGATACGACAAAGAGTTACTCTCGAAGGTCTGGAGTTAGAACCAGAAGAAACTCGAAGAGAAAGCTCGTGAGAAGGCTCTGGCTGATGTGAACAAGCGAGCACCTATAGTTGCCGCTACGAAGGCCCGTGAAGCTGCTGCTAAACGAGACCGAGAGAAACGTAAACAGACTCACAAGTTCATCTACGGACGTAAGGAGTAAATAAAGGTTACATaa                                                                                                                                                                                                                                                                                                       >  1:216720/1‑301 (MQ=255)`  
     `cccTTGGATAACCAATAAGCAACTGACCATGTATTCTCAAGGTGACTCCATTTACCTTATGGACACCACAGGTCAAGTCAGAGTCCGATACGACAAAGAGTTACTCTCGAAGGTCTGGAGTGAGAACCAGAAGAAACTCGAAGAGAAAGCTCGTGAGAAGGCTCTGGCTGATGTGAACAAGCGAGCACCTATAGTTGCCGCTACGAAGGCCCGTGAAGCTGCTGCTAAACGAGTCCGAGAGAAACGTAAACAGACTCCTAAGTTCATCTACGGACGTAAGGAGTAACTAAAGGTTACAtct                                                                                                                                                                                                                                                                                                       >  1:119048/1‑299 (MQ=255)`  
       `caaggAGAACCAATAAGCAACTGAACATGTAATCACAAGGTGACTACATATACCTGAAGGAAACCAAAGGTAAAGGCAGAGTCCGATACGACAAAGAGTGACGCGAGAAGGTAAGGAGGGAGAACGAGAAGAAACTCGAAGAGAAAGCTCGTGAGAAGGATCTGGATGAAGGGAACAAGCGAGAAAAAATAGTTGGCGAAAAGAAGGCCGGTGAAGCTGATGCTAAAAGAGTACGAGATAAACGTAAACAGACTCCTAAGTTCATCTACGGACGTAAGGAGTAACTAAAGGTTACATAagg                                                                                                                                                                                                                                                                                                     <  1:100073/298‑1 (MQ=255)`  
        `gtgtataagagacaggCAACTGACCATGTATTCTCAAGGTGACTCCATTTACCTTATGGACACCACAGGTCAAGTCAGAGTCCGATACGACAAAGAGTTACTCTCGAAGGTCTGGAGTGAGAACCAGAAGAAACTCGAAGAGAAAGCTCGTGAGAAGGCTCTGGCTGATGTGAACAAGCGAGCACCTATAGTTGCCGCTACGAAGGCCCGTGAAGCTGCTGCTAAACGAGTCCGAGAGAAACGTAAACAGACTCCTAAGTTCATCTACGGACGTAAGGAGTAACTAAAGGCTACATAagga                                                                                                                                                                                                                                                                                                    <  1:258553/286‑1 (MQ=255)`  
         `tGGATAACCAATAAGCAACTGACCATGTATTCTCAAGGTGACTCCATTTACCTTATGGACACCACAGGTCAAGTCAGAGTCCGATACGACAAAGAGTTACTCTCGAAGGTCTGGAGTGAGAACCAGAAGAAACTCGAAGAGAAAGCTCGTGAGAAGGCTCTGGCTGATGTGAACAAGCGAGCACCTATAGTTGCCGCTACGAAGGCCCGTGAAGCTGCTGCTAAACGAGTCCGAGAGAAACGTAAACAGACTCCTAAGTTCATCTACGGACGTAAGGAGTAACTAAAGGTTACATAaggag                                                                                                                                                                                                                                                                                                   <  1:249226/301‑1 (MQ=255)`  
               `aCCAATAAGCAACTGACCATGTATTCTCAAGGTGACTCCATTTACCTTATGGACACCACAGGTCAAGTCAGAGTCCGATACGACAAAGAGTTACTCTCGAAGGTCTGGAGTGAGAACCAGAAGAAACTCGAAGAGAAAGCTCGTGAGAATGCTCTGGCTGATGTGAACAAGCGAGCACCTATAGTTGCCGCTACGAAGGCCCGTAAAGCTGCTGCTAAACGAGTCCGAGAGAAACGTAAACAGACTCCTAAGTTCATCTACGGACGTAAGGAGTAACGAAAGGTTACAGAAGGAGGCCCTa                                                                                                                                                                                                                                                                                             >  1:82927/1‑301 (MQ=255)`  
               `aCCAATAAGCAACTGACCATGTATTCTCAAGGTGACTCCATTTACCTTATGGACACCACAGGTCAAGTCAGAGTCCGATACGACAAAGAGTTACTCTCGAAGGTCTGGAGTGAGAACCAGAAGAAACTCGAAGAGAAAGCTCGTGAGAAGGCTCTGGCTGATGTGAACAAGCGAGCACCTATAGTTGCCGCTACGAAGGCCAGTGAAGCTGCTGCTAAACGAGTCCGAGAGAAACGTAAACAGACTCCTAAGTTCATCTACGGACGTAAGGAGTAACTAAAGGCTACATAAGGAGGCCCTa                                                                                                                                                                                                                                                                                             >  1:132881/1‑301 (MQ=255)`  
                           `cTGACCATGTATTCTCAAGGTGACTCCATTTACCTTATGGACACCACAGGTCAAGTCAGAGTCCGATACGACAAAGAGTTACTCTCGAAGGTCTGGAGTGAGAACCAGAAGAAACTCGAAGAGAAAGCTCGTGAGAAGGCTCTGGCTGATGTGAACAAGCGAGCACCTATAGTTGCCGCTACGAAGGCCCGTGAAGCTGCTGCTAAACGAGTCCGAGAGAAACGTAAACAGACTCCTAAGTTCATCTACGGACGTAAGGAGTAACTAAAGGCTACATAAGGAGGCCCTAAATGGATAAGTa                                                                                                                                                                                                                                                                                 >  1:163728/1‑301 (MQ=255)`  
                             `gACCATGTATTCTCAAGGTGACTCCATTTACCTTATGGACACCACAGGTCAAGTCAGAGTCCGATACGACAAAGAGTTACTCTCGAAGGTCTGGAGTGAGAACACGAAGAAACTCGAAGAGAAAGCTCGTGACAAGGCTCTGGCTGATGTGAACAAGCGAGCACCTATAGTTGCCGCTACCAAGGCCCGTGAAGCTTCTGCTAAACGAGTCCGAGAGAAACGTAAACAGACTCCTAAGTTCATCTACGGACGTAAGGAGTAACTAAATGCTACATAAGGAGGACCTAAATGGATAAGTACg                                                                                                                                                                                                                                                                               >  1:246947/1‑301 (MQ=255)`  
                              `aCCATGTATTCTCAAGGTGACTCCATTTACCTTATGTACACCACAGGTCAAGTCAGAGTCGGATACGACAAAGAGTTACTCTTGAAGGTCTGGAGTGAGAACCAGAAGAAACTCGAAGAGTAAACTCGTGAGAAGTATCTGGCTGATGTGAACAAGCGAGCACATATAGTTGCCGCTACGAAGGCCCGTGAATCTGATGCTAAACGAGTCCGAGAGAAACGTAAACAGACTCCTAAGTTCATCTACGGTCATAATGAGTAATTAAAGGTTACATAAGAAGGCCCTAAATGGagactgtctt                                                                                                                                                                                                                                                                              >  1:234895/1‑292 (MQ=255)`  
                                `cATGTATTCTCAAGGTGAATCCATTTACCTTATGGACACCACAGGTCAAGTCAGAGTCCGATACGACAAAGAGTTACTCTCGAAGGTCTGGAGTGAGAACCAGAAGAAACTCGAAGAGAAAGCTCGTGAGAAGGCTCTGGCTGATGTGAACAAGCGAGCACCTATAGTTGCCGCTACGAAGGCCCGTGAAGCTGCTGCTAAACGAGTCCGAGAGAAACGTAAACAGACTCCTAAGTTCATCTACGGACGTAAGGAGTAACTAAAGGCTACATAAGGAGGCCCTAAATGGATAAGTACGATa                                                                                                                                                                                                                                                                            <  1:40534/301‑1 (MQ=255)`  
                                   `gTATTCTCAAGGTGACTCCATTTACCTTATGGACACCACAGGTCAAGTCAGAGTCCGATACGACAAAGAGTTACTCTCGAAGGTCTGGAGTGAGAACCAGAAGAAACTCGAAGAGAAAGCTCGTGAGAAGGCTCTGGCTGATGTGAACAAGCGAGCACCTATAGTTGCCGCTACGAAGGCCCGTGAAGCTGCTGCTAAACGAGTCCGAGAGAAACGTAAACAGACTCCTAAGTTCATCTACGGACGTAAGGAGTAACTAAAGGTTACATAAGGCGGCCCTAAAtctgtctcttatacacat                                                                                                                                                                                                                                                                         >  1:56919/1‑284 (MQ=255)`  
                                   `gTATTCTCAAGGTGACTCCATTTACCTTATGGACACCACAGGTCAAGTCAGAGTCCGATACGACAAAGAGTTACTCTCGAAGGTCTGGAGTGAGAACCAGAAGAAACTCGAAGAGAAAGCTCGTGAGAAGGCTCTGGCTGATGTGAACAAGCGAGCACCTATAGTTGCCGCTACGAAGGCCCGTGAAGCTGCTGCTAAACGAGTCCGAGAGAAACGTAAACAGACTCCTAAGTTCATCTACGGACGTAAGGAGTAACTAAAGGTTACATAAGGAGGCCCTAAATGGATAAGTACGATAAGa                                                                                                                                                                                                                                                                         >  1:61898/1‑301 (MQ=255)`  
                                    `tgtgctaaagggaattCCATTGACCTTAGTGACACCACAGGTCAAGTCAGAGACCGATACGACAAAGAGTTACACGCGAAGGTCTGGAGTGAGAACCAGAAGAAACTCGAAGAGAAAGCGCGTGAGAAGGCTCTGGCTGATGTGAACAAGCGAGCACCTATAGTTGCCGCTACGAAGGCCCGTGAAGCTGCTGCTAAACGAGGCCGAGAGAAACGTAAACAGACGCCTAAGTTCATCTACGGACGTAAGTAGTAACTAAAGGCTACATAAGGAGGCCCTAAATGGATAAGTACGATAAGaa                                                                                                                                                                                                                                                                        <  1:37343/286‑1 (MQ=255)`  
                                      `ttCTCAAGGTGACTCCATTTACCTTATGGACACCACAGGTCAAGTCAGAGTCCGATACGACAAAGAGTTACTCTCGAAGGTCTGGAGTGAGAACCAGAAGAAACTCGAATAGAAAGCTCGTGAGAAGGCTCTGGCTGATGTGAACAAGCGAGCACCTATAGTTGCCGCTACGAAGGCCCGTGAAGCTGCTGCTAAACGAGTCCGAGAGAAACGTAAACAGACTCCTAAGTTCATCTACGGACGTAAGGAGTAACTAAAGGCTACATAAGGAGGCCCTAAATGGATAAGTACGATAAGAACg                                                                                                                                                                                                                                                                      >  1:283302/1‑301 (MQ=255)`  
                                       `tctcAAGGTGACTCCATTTACCTTATGGACACCACAGGTCAAGTCAGAGTCCGATACGACAAAGAGTTACTCTCGCAGGTCTGGAGTGAGAACCAGAAGAAACTCGAAGAGAAAGCTCGTGAGAAGGCTCTGGCTGATGTGAACAAGCGAGCACCTATAGTTGCCGCTACGAAGGCCCGTGAAGCTGCTGCTAAACGAGTCCGAGAGAAACGTAAACAGACTCCTAAGTTCATCTACGGACGTAAGGCGTAACTAAAGGCTACATAAGGAGGCCCTAAATGGATAAGTACGATAAGAACGt                                                                                                                                                                                                                                                                     >  1:181520/1‑301 (MQ=255)`  
                                         `ctcggagatgtgtataagagacagggAAACCACAGGTCAAGTCAGAGTCCGATACGACAAAGAGTTACTCTCGAAGGTCTGGAGTGAGAACCAGAAGAAACTCGAAGAGAAAGCTCGTGAGAAGGCTCTGGCTGATGTGAACAAGCGAGCACCTATAGTTGCCGCTACGAAGGCCCGTGAAGCTGCTGCTAAACGAGTCCAAGAGTAACGTAAACAGACTCCTAAGTTCATCTACGGACGTAAGGAGTAACTAAAGGCTACATAAGGAGGCCCTAAATGGATAAGTACGATAAGAACGTAc                                                                                                                                                                                                                                                                   <  1:120614/277‑1 (MQ=255)`  
                                          `cAAGGTGACTCCATTTACCTTATGGACACCACAGGTCAAGTCAGAGTCCGATACGACAAAGAGTTACTCTCGAAGGTCTGGAGTGAGAACCAGAAGAAACTCGAAGAGAAAGCTCGTGAGAAGGCTCTGGCTGATGTGAACAAGCGAGCACCTATAGTTGCCGCTACGAAGGCCCGTGAAGCTGCTGCTAAACGAGTCCGAGAGAAACGTAAACAGACTCCTAAGTTCATCTACGGACGTAAGGAGTAACTAAAGGCTACATAAGGAGCCCCTAAATGGATAAGTACGATAAgctgtctct                                                                                                                                                                                                                                                                  >  1:43381/1‑293 (MQ=255)`  
                                            `aGGTGACTCCATTTTCCTTATGGACACCACAGGTCAAGTCAGAGTCCGATACGACAAAGAGTTAATCTCGAAGGTCTGGAGTGAGAACCAGAAGAAACTCGAAGAGAAAGCTCGTGAGAAGGCTCTGGCTGATGTGAACAAGCGAGCACCTATAGTTGCCGCTACGAAGGCCCGTGAAGCTGCTGCTAAACGAGTCCGAGAGAAACGTAAACAGACTCCTAAGTTCATCTACGGACGTAAGGAGTAACTAAAGGCTACATAAGGAGGCCCTAAATGGATAAGTACGATAAgctgcctctta                                                                                                                                                                                                                                                                >  1:279984/1‑291 (MQ=255)`  
                                            `aGGTGACTCCATTTACCTTATGGACACCACAGGTCAAGTCAGAGTCCGATACGACAAAGAGTTACTCTCGAAGGTCTGGAGTGAGAACCAGAAGAAACTCGAAGAGAAAGCTCGTGAGAAGGCTCTGGCTGATGTGAACAAGCGAGCACCTATAGTTGCCGCTACGAAGGCCCGTGAAGCTGCTGCTAAACGAGTCCGAGAGAAACGTAAACAGACTCCTAAGTTCATCTACGGACGTAAGGAGTAACTAAAGGCTACATAAGGAGGCCCTAAATGGATAAGTACGATAAGAACGTACCaa                                                                                                                                                                                                                                                                >  1:210189/1‑301 (MQ=255)`  
                                               `gtgtataagagacagtagggACACCACAGGTCAAGTCAGAGTCCGATACGACAAAGAGTTACTCTCGAAGGTCTGGAGTGAGAACCAGAAGAAACTCGAAGAGAAAGCTCGTGAGAAGGCTCTGGCTGATGTGAACAAGCGAGCACCTATAGTTGCCGCTACGAAGGCCCGTGAAGCTGCTGCTAAACGAGTCCGAGAGAAACGTAAACAGACTCCTAAGTTCATCTACGGACGTAAGGAGTAACTAAAGGTTACATAAGGAGGCCCTAAATGGATAAGTACTATAAGAACGTACCAAGTg                                                                                                                                                                                                                                                             <  1:69380/283‑1 (MQ=255)`  
                                               `ataaaagacagaCCTAATGGACACCACATGTCAAGTCAGAGTCCGATACGACAAAGAGTTACTCTCGAAGGTCTGGAGTGAGAACCAGAAGAAACTCGAAGAGAAAGCTCGTGAGAAGGCTCTGGCTGATGTGAACAAGCGAGCACCTATAGTTGCCGCTACGAAGGCCCGTGAAGCTGCTGCTAAACGAGTCCGAGAGAAACGTAAACAGACTCCTAAGTTCATCTACGGACGTAAGGAGTAACTAAAGGCTACATAAGGAGGCCCTAAATGGATAAGTACGATAAGAACGTACCAAGTg                                                                                                                                                                                                                                                             <  1:35645/290‑1 (MQ=255)`  
                                                  `cTCCATTTACCTTATGTACACCACAGGTCAAGTCAGAGTCCGATACGACAAAGAGTTACTCTCGAAGGTCTGGAGTGAGAACCAGAAGAAACTCGAAGAGAAAGCTCGTGAGAAGGCTCTGGCTGATGTGAACAAGCGAGCACCTATAGTTGCCGCTACGAAGGCCCGTGAAGCTGCTGCTAAACGAGTCCGAGAGAAACGTAAACAGACTCCTAAGTTCATCTACGGACGTAAGGAGTAACTAAAGGTTACATAATAAGGACCTAAATGGATAAGTACGATAAGAACGTATCAAgcgctg                                                                                                                                                                                                                                                          >  1:196693/1‑296 (MQ=255)`  
                                                  `cTCCATTTACCTTATGGACACCACAGGTCAAGTCAGAGTCCGATACGACAAAGAGTTACTCTCGAAGGTCTGGAGTGAGAACCAGAAGAAACTCGAAGAGAAAGCTCGTGAGAAGGCTCTGGCTGATGTGAACAAGCGAGCACCTATAGTTGCCGCTACGAAGGCCCGTGAAGCTGCTGCTAAACGAGTCCGAGAGAAACGTAAACAGACTCCTAAGTTCATCTACGGACGTAAGGAGTAACTAAAGGTTACATAAGGAGGCCCTAAATGGATAAGTACGATAAGAACGTACCAAGTGAtt                                                                                                                                                                                                                                                          >  1:135636/1‑301 (MQ=255)`  
                                                    `ccATTTACCTTATGGACACCACAGGTCAAGTCAGAGTCCGATACGACAAAGAGTTACTCTCGAAGGTCTGGAGTGAGAACCAGAAGAAACTCGAAGAGAAAGCTCGTGAGAAGGCTCTGGCTGATGTGAACAAGCGAGCACCTATAGTTGCCGCTACGAAGGCCCGTGAAGCTGCTGCTAAACGAGTCCGAGAGAAACGTAAACAGACTCCTAAGTTCATCTACGGACGTAAGGAGTAACTAAAGGTTACATAAGGAGGCCCTAAATGGATAAGTACGATAAGAACGTACCAAGTGATTaa                                                                                                                                                                                                                                                        >  1:315847/1‑300 (MQ=255)`  
                                                    `ccATTTACCTTATGGACACCACAGGTCAAGTCAGAGTCCGATACGACAAAGAGTTACTCTCGAAGGTCTGGAGTGAGAACCAGAAGAAACTCGAAGAGAAAGCTCGTGAGAAGGCTCTGGCTGATGTGAACAAGCGAGCACCTATAGTTGCCGCTACGAAGGCCCGTGAAGCTGCTGCTAAACGAGTCCGAGAGAAACGTAAACAGACTCCTAAGTTCATATACGGACGTAAGGAGTAAATAAAGGTTACATAAGGAGGCCCTAAATGGATAAGTACGATAAGAACGTACCAAGTGATTag                                                                                                                                                                                                                                                        >  1:54277/1‑300 (MQ=255)`  
                                                    `acATTTACCTTATGGACACCACAGGTCAAGTCAGAGTCAGATACGAAAAAGAGTGAATCTCGAATGTATGGAGTGAGAACCAGAAGAAACTCGAAGAGAAAGCTCGTGAGAAGGCTCTGGCTGATGTGAACAAGCGAGCACCTATAGTTGCCGCTACGAAGGCCCGTGAAGCTGCTGCTAAACGAGTCCGAGAGAAACGTAAACAGACTCCTAAGTTCATCTACGGACGTAAGGAGTAACTAAAGGCTACATAAGGAGGCCCTAATTGGATAAGTACGATAAGAACGTACCAAGTGATTat                                                                                                                                                                                                                                                        <  1:18923/300‑1 (MQ=255)`  
                                                       `tttACCTTATGGACACCACAGGTCAAGTCAGAGTCCGATACGACAAAGAGTTACTCTCGAAGGTCTGGAGTGAGAACCAGAAGAAACTCGAAGAGAAAGCTCGTGAGAAGGCTCTGGCTGATGTGAACAAGCGAGCACCTATAGTTGCCGCTACGAAGGCCCGTGAAGCTGCTGCTAAACGAGTCCGAGAGAAACGTAAACAGACTCCTAAGTTCATCTACGGACGTAAGGAGTAACTAAAGGTTACATAAGGAGGCCCTAAATGGATAAGTACGATAAGAACGTACCAATTGATTatgat                                                                                                                                                                                                                                                     <  1:339641/301‑1 (MQ=255)`  
                                                       `gagatgtgtataagagacagggTCAAGTCAGAGTCCGATACGACAAAGAGTTACTCTCGAAGGTCTGGAGTGAGAACCAGAAGAAACTCGAAGAGAAAGCTCGTGAGAAGGCTCTGGCTGATGTGAACAAGCGAGCACCTATAGTTGCCGCTACGAAGGCCCGTGAAGCTGCTGCTAAACGAGTCCGAGAGAAACGTAAACAGACTCCTAAGTTCATCTACGGACGTAAGGAGTAACTAAAGGCTACATAAGGAGGCCCTAAATGGATAAGTACGATAAGAACGTACCAAGTGATTatgat                                                                                                                                                                                                                                                     <  1:78763/281‑1 (MQ=255)`  
                                                       `aagaCCTAAGGGACACAAAAGGTCAAGTCAGAGTCCGATACGACAAAGAGTTACGCTCGAAGGTCTGGAGTGAGAACCAGAAGAAACTCGAAGAGAAAGCTCGTGAGAAGGCTCTGGCTGATGTGAACAAGCGAGCACCTATAGTTGCCGCTACGAAGGCCCGTGAAGCTGCTGCTAAACGAGTCCGAGAGAAACGTAAACAGACTCCTAAGTTCATCTACGGACGTAAGGAGTAACTAAAGGCTACATAAGGAGGCCCTAAATGGATAAGTACGATAAGAACGTACCAAGTGATTatgat                                                                                                                                                                                                                                                     <  1:318749/298‑1 (MQ=255)`  
                                                         `tACCTTATGGACACCACAGGTCAAGTCAGAGTCCGATACGACAAAGAGTTACTCTCGAAGGTCTGGAGTGAGAACCAGAAGAAACTCGAAGAGAAAGCTCGTGAGAAGGCTCTGGCTGATGTGAACAAGCGAGCACCTATAGTTGCCGCTACGAAGGCCCGTGAAGCTGCTGCTAAACGAGTCCGAGAGAAACGTAAACAGACTCCTAAGTTCATCTACGGACGTAAGGAGTAACTAAAGGCTACATAAGGAGGCCCTAAATGGATAAGTACGATAAGAACGTACCAAGTGAGTATGATgg                                                                                                                                                                                                                                                   <  1:186188/301‑1 (MQ=255)`  
                                                         `gtataagagacagccacAGGTCAAGTCAGAGTCCGATACGACAAAGAGTTACTCTCGAAGGTCTGGAGTGAGAACCAGAAGAAACTCGAAGAGAAAGCTCGTGAGAAGGCTCTGGCTGATGTGAACAAGCGAGCACCTATAGTTGCCGCTACGAAGGCCCGTGAAGCTGCTGCTAAACGAGTCCGAGAGAAACGTAAACAGACTCCTAAGTTCATCTACGGACGTAAGGAGTAACTAAAGGTTACATAAGGAGGCCCTAAATGGATAAGTACGATAAGAACGTACCAAGTGATTATGATgg                                                                                                                                                                                                                                                   <  1:72683/288‑1 (MQ=255)`  
                                                          `aCCTTATGGACACCACATGTCAAGTCAGAGTCCGATACGACAAAGAGTTACTCTCGAAGGTCTGGAGTGAGAACCAGAAGAAACTCGAAGAGAAAGCTCGTGAGAAGGATCTGGCTGATGTGAACAAGCGAGCACCTATAGTTGCCGCTACGAAGGCCCGTGAAGCTGCTGCTAAACGAGTCCGAGAGAAACGTAAACAGACTCCTAAGTTCATCTACGGACGTAAGGAGTAACTAAAGGTTACATAAGGAGGCCCTAAATGGATAAGTACGATAAGAACGTACCAAGTGATTactgtctc                                                                                                                                                                                                                                                  >  1:220017/1‑294 (MQ=255)`  
                                                           `ccTTATGGACACCACAGGTCAAGTCAGAGTCCGATACGACAAAGAGTTACTCTCGAAGGTCTGGAGTGAGAACCAGAAGGAACTCGAAGAGAAAGCTCGTGAGAAGGCTCTGGCTGATGTGAACAAGCGAGCACCTATAGTTGCCGCTACGAAGGCCCGTGAAGCTGCTGCTAAACGAGTCCGAGAGAAACGTAAACAGACTCCTAAGTTCATCTACGGACGTTAGTAGTAACTAAAGGTTACATAAGGAGGCCCTAAATGGATAAGTACGATAAGAACGTACCAAGTGATTATGATGGTc                                                                                                                                                                                                                                                 <  1:145878/301‑1 (MQ=255)`  
                                                           `ccTTATGGACACCACAGGTCAAGTCAGAGTCCGATACGACAAAGAGTTACTCTCGAAGGTCTGGAGTGAGAACCAGAAGAAACTCGAAGAGAAAGCTCGTGAGAAGGCTCTGGCTGATGTGAACAAGCGAGCACCTATAGTTGCCGCTACGAAGGCCCGTGAAGCTGCTGCTAAACGAGTCCGAGAGAAACGTAAACAGACTCCTAAGTTCATCTACGGACTTAAGGAGTAACTAAAGGTTACATAAGGAGGCCCTAAATGGATAAGTACGATAAGAACGTACCAAGTGATTATGATGGTc                                                                                                                                                                                                                                                 >  1:228626/1‑301 (MQ=255)`  
                                                           `ccTTATGGACACCACAGGTCAAGTCAGAGTCCGATACGACAAAGAGTTACTCTCGAAGGTCTGGAGTGAGAACCAGAAGAAACTCGAAGAGAAAGCTCGTGAGAAGGCTCTGGCTGATGTGAACAAGCGAGCACCTATAGTTGCCGCTACGAAGGCCCGTGAAGCTGCTGCTAAACGAGTCCGAGAGAAACGTAAACAGACTCCTAAGTTCATCTACGGACGTAAGGAGTAACTAAAGGCTACATAAGGAGGCCCTAAATGGATAAGTACGATAAGAACGTACCAAGTGATTATGATGGtt                                                                                                                                                                                                                                                 >  1:179496/1‑300 (MQ=255)`  
                                                           `ccTTATGGACACCACAGGTCAAGTCAGAGGCCGATACGACAAAGAGTTACTCTCGAAGGTCTGGAGTGAGAACCAGAAGAAACTCGAAGAGAAAGCTCGTGAGAAGGCTCTGGATTATGTGAACAAGCGAGAACCTATAGTTGCCGCTACGAAGGCCCGTGAAGCTGCTGCTAAACGAGTCCGAGAGAAACGTAAACAGACTCCTAAGTTCATCTACGGACGTAAGGAGTAACTAAAGGCTACATAAGGAGGCCCTAAATGGATAAGTACGATAAGAACGTACCAAGTGATTATGATGGTc                                                                                                                                                                                                                                                 <  1:261967/301‑1 (MQ=255)`  
                                                           `ccTTATGGACACCACAGGTAAAGTCAGAGTCCGATACGACAAAGAGTTACTCTCGAAGGTCTGGAGTGAGAACCAGAAGAAACTCGAAGAGAAAGCTCGTGAGAAGGCTCTGGCTGATGTGAACAAGCGAGCACCTATAGTTGCCGCTACGAAGGCCCGTGAAGCTGCTGCTAAACGAGTCCGAGAGAAACGTAAACAGACTCCTAAGTTCATCTACGGACGTAAGGAGTAACTAAAGGTTACATAAGGAGGCCCTAAATGGATAAGTACGATAAGAACGTACCAAGTGATTATGATGGTc                                                                                                                                                                                                                                                 <  1:222913/301‑1 (MQ=255)`  
                                                                  `gACACCACAGGTCAAGTCAGAGTCCGATACGACAAAGAGTTACTCTCGAAGGTCTGGAGTGAGAACAAGAAGAAACTCGAAGAGAAAGCTCGTGAGAAGGCTCTGGCTGATGTGAACAAGCGAGCACCTATAGTTGCCGCTACGAAGGCCCGTGAAGCTGCTGCTAAACGAGTCCGAGAGAAACGTAAACAGACTCCTAAGTTCATCTACGGACGTAAGGAGTAACTAAAGGCTACATAAGGAGGCCCTAAATGGATAAGTACGATAAGAACGTACCAAGTGATTATGATGGTCTGTTCCa                                                                                                                                                                                                                                          >  1:95511/1‑301 (MQ=255)`  
                                                                     `accacAGGTCAAGTCAGAGTCCGATACGACAAAGAGTTACTCACGAAGGTATGGAGTGAGAACCAGAAGAAACTCGAAGAGAAAGCTCGTGAGAAGGCTCTGGCTGATGTGAACAAGCGAGCACCTATAGTTGCCGCTACGAAGGCCAGTGAAGCTGCTGCTAAACGAGTCCGAGAGAAACGTAAACAGACTACTAAGTACAGCTACGGACGTAAGGAGTAACTAAAGGTTACATAAGGAGGCCCTAAATGGATAAGTACGATAAGAACGTACCAAGTGATTATGATGGTCTGTTCCaaaa                                                                                                                                                                                                                                       <  1:52644/301‑1 (MQ=255)`  
                                                                                 `gTCAGAGTCCGATACGACAAAGAGTTACTCTCGAAGGTCTGGAGTGAGAACCAGAAGAAACTCGAAGAGAAAGCTCGTGAGAAGGCTCTGGCTGATGTGAACAAGCGAGCACCTATAGTTGCCGCTACGAAGGCCCGTGAAGCTGCTGCTAAACGAGTCCGAGAGAAACGTAAACAGACTCCTAAGTTCATCTACGGACGTAAGGAGTAACTAAAGGTTACATAAGGAGGCCCTAAATGGATAAGTACGATAAGAACGTACCAAGTGATTATGACTGTCTCttatacacatctccgagccc                                                                                                                                                                                                                           >  1:150425/1‑283 (MQ=255)`  
                                                                                 `gTCAGAGTCCGATACGACAAAGAGTTACTCTCGAAGGTCTGGAGTGAGAACCAGAAGAAACTCGAAGAGAAAGCTCGTGAGAAGGCTCTGGCTGATGTGAACAAGCGAGCACCTATAGTTGCCGCTACGAAGGCCCGTGAAGCTGCTGCTAAACGAGTCCGAGAGAAACGTAAACAGACTCCTAAGTTCATCTACGGACGTAAGGAGTAACTAAAGGCTACATAAGGAGGCCCTAAATGGATAAGTACGATAAGAACGTACCAAGTGATTATGCTGTTCTGTTCCAAAAGGCTGCTGATGc                                                                                                                                                                                                                           >  1:92873/1‑301 (MQ=255)`  
                                                                                 `gTCAGAGTCCGATACGACAAAGAGTTACTCTCGAAGGTCTGGAGTGAGAACCAGAAGAAACTCGAAGAGAAAGCTCGTGAGAAGGCTCTGGCTGATGTGAACAAGCGAGCACCTATAGTTGCCGCTACGAAGGCCCGTGAAGCTGCTGATAAACGAGTCCGAGAGAAACGTAAACAGACTCCTAAGTTCATCTACGGACGTAAGGAGTAACTAAAGGCTACATAAGGAGGCCCTAAATGGATAAGTACGATAAGAACGTACCAAGTGATTATGATGGTCTGTTCCAAAAGGCTGCTGATGc                                                                                                                                                                                                                           >  1:334889/1‑301 (MQ=255)`  
                                                                                 `gTCAGAGTCCGATACGACAAAGAGTTACTCTCGAAGGTCTGGAGTGAGAACCAGAAGAAACTCGAAGAGAAAGCTCGTGAGAAGGCTCTGGCCGATGTGAACAATCGAGCACCTATAGTTGCCGCTACGGAGGCCCTTGAAGCTGCTGCTAAACGAGTCCGAGAGAAACGTAAACAGACTCCTACGTTCATCTACGGACGTAAGGAGTAACTAAAGGTTACATAAGGAAGCCCTACATGGATAAGTACGATAAGAACGTACCAAGTGATTATGATGGTCTGTTCCAAAAGGCTGCTGATGc                                                                                                                                                                                                                           >  1:155639/1‑301 (MQ=255)`  
                                                                                         `ccGATACGACAAAGAGTTACTCTCGAAGGTCTGGAGTGAGAACCAGAAGAAACTCGAAGAGAAAGCTCGTGAGAAGGCTCTGGCTGATGTGAACAAGCGAGCACCTATAGTTGCCGCTACGAAGGCCCGTGAAGCTGCTGCTAAACGAGTCCGAGAGAAACGTAAACAGACTCCTAAGTTCATCTACGGACGTAAGGAGTAACTAAAGGTTACATAAGGAGGCCCTAAATGGATAAGTACGATAAGAACGTACCAAGTGATTATGATGGTCTGTTCCAAAAGGCTGCTGATGCCAACgggt                                                                                                                                                                                                                   >  1:213105/1‑300 (MQ=255)`  
                                                                                                `gagatgtgtataagagacagggTCTGGAGTGAGAACCAGAAGAAACTCGAAGAGAAAGCTCGTGAGAAGGCTTTGGCTGATGTGAACAAGCGAGCACCTATAGTTGCCGCTACGAAGGCCCGTGAAGCTGCTGCTAAACGAGTCCGAGAGAAACGTAAACAGACTCCTAAGTTCATCTACGGACGTAAGGAGTAACTAAAGGCTACATAAGGAGGCCCTAAATGGATAAGTACGATAAGAACGTACCAAGTGATTATGATGGTCTGTTCCAAAAGGCTGCTGATGCCAACGGGGTCTCTTa                                                                                                                                                                                                            <  1:176992/281‑1 (MQ=255)`  
                                                                                                `gACAAAGAGGTACTCGCGAAGGTCTGGAGTGAGAACCAGAAGAAACTCGAAGAGAAAGCTCGTGAGAAGACTCCGGCTGATGTGAACAAGCGAGCACCTATAGTTGCCGCTACGAAGGCCCGTGAAGCTGCTGCTAAACGAGTCCGAGAGAAACGTAAACAGACTCCTAAGTTCATCTACGGACGTAAGGAGTAACTAAAGGCTACATAAGGAGGCCCTAAATGGATAAGTACGATAAGAACGTACCAAGTGATTATGATGGTCTGTTCCAAAAGGCTGCTGATGCCAACGGGGTCTCTTa                                                                                                                                                                                                            <  1:299456/301‑1 (MQ=255)`  
                                                                                                   `aaaGAGTTACTCTCGAAGGTCTGGAGTGAGAACCAGAAGAAACTCGAAGAGAAAGCTCGTGAGAAGGCTCTGGCTGATGTGAACAAGCGAGCACCTATAGTTGCCGCTACGAAGGCCCGTGAAGCGGCTGCTAAACGAGTCCGAGAGAAACGTAAACAGACTCCTAAGTTCATCTACGGACGTAAGGAGTAACTAAAGGTTACATAAGGAGGCCCTAAATGGATAAGTACGATAAGAACGTACCAAGTGATTATGATGGTCTGTTCCAAAAGGCTGCTGATGCCAACGGGGTCTCTTATGa                                                                                                                                                                                                         <  1:269082/301‑1 (MQ=255)`  
                                                                                                    `acaggTTACTCTCGAAGGTCTGGAGTGAGAACCAGAAGAAACTCGAAGAGAAAGCTCGTGAGAAGGCTCTGGCTGATGTGAACAAGCGAGCACCTATAGTTGCCGCTACGAAGGCCAGTGAAGCTGCTGCTAAACGAGTCCGAGAGAAACGTAAACAGACTCCTAAGTTCATCTACGGACGTAAGGAGTAACTAAAGGCTACATAAGGAGGCCCTAAATGGATAAGTACGATAAGAACGTACCAAGTGATTATGATGGTCTGTTCCAAAAGGCTGCTGATGCCAACGGGGTCTCTTATGAc                                                                                                                                                                                                        <  1:255012/297‑1 (MQ=255)`  
                                                                                                     `gtgtataagagacagggTCTGGAGTGAGAACCAGAAGAAACTCGAAGAGAAAGCTCGTGAGAAGGCTCTGGCTGATGTGAACAAGCGAGCACCTATAGTTGCCGCTACGAAGGCCCGTGAAGCTGCTGCTAAACGAGTCCGAGAGAAACGTAAACAGACTCATAAGTTCATCTACGGACGTAAGGAGTAACTAAAGGCTACATAAGGAGGCCCTAAATGGATAAGTACGATAAGAACGTACCAAGTGATTATGATGGTCTGTTCCAAAAGGCTGCTGATGCCAACGGGGTCTCTTATGAcc                                                                                                                                                                                                       <  1:276142/286‑1 (MQ=255)`  
                                                                                                     `ggagTTACTCTCGAAGGTCTGGAGTGAGAACCAGAAGAAACTCGAAGAGAAAGCTCGTGAGAAGGCTCTGGCTGATGTGAACAAGCGAGCACCTATAGTTGCCGCTACGAAGGCCCGTGAAGCTGCTGCTAAACGAGTCCGAGAGAAACGTAAACAGACTCCTAAGTTCATCTACGGACGTAAGGAGTAACTAAAGGTTACATAAGGAGGCCCTAAATGGATAAGTACGATAAGAACGTACCAAGTGATTATGATGGTCTGTTCAAAAAGGCTGCTGATGCCAACGGGGTCTCTTATGAcc                                                                                                                                                                                                       <  1:306643/300‑1 (MQ=255)`  
                                                                                                     `agagTTACTCTCGAAGGTCTGGAGTGAGAACCAGAAGAAACTCGAAGAGAAAGCTCGTGAGAAGGCTCTGGCTGATGTGAACAAGCGAGCACCTATAGTTGCCGCTACGAAGGCCCGTGAAGCTGCTGCTGAACGAGTCCGAGAGAAACGTAAACAGACTCCTAAGTTCATCTACGGACGTAAGGAGTAACTAAAGGTTACATAAGGAGGCCCTAAATGGATAAGTACGATAAGAACGTACCAAGTGATTATGATGGTCTGTTCCAAAAGGCTGCTGATGCCAACGGGGTCTCTTATGAcc                                                                                                                                                                                                       <  1:232394/301‑1 (MQ=255)`  
                                                                                                     `agagTTACTCTCGAAGGTCTGGAGTGAGAACCAGAAGAAACTCGAAGAGAAAGCTCGTGAGAAGGCTCTGGCTGATGTGAACAAGCGAGCACCTATAGTTGCCGCTACGAAGGCCCGTGAAGCTGCTGCTAAACGAGTCCTAGAGAAACGTAAACAGACTCCTAAGTTCATCTACTGACGTAAGGAGTAACTAAAGGCTACATAAGGAGGCCCTAAATGGATAAGTACGATAAGAACGTACCAAGTGATTATGATGGTCTGTTCCAAAAGGCTGCTGATGCCAACGGGGTATCTTATGAcc                                                                                                                                                                                                       <  1:130863/301‑1 (MQ=255)`  
                                                                                                             `ccacGAAGGTCTGGAGTGAGAACCAGAAGAAACTCGAAGATAAATCTCGTGAGAAGGCTCTGGCTGATGTGAACAAGCGAGCACCTATAGTTGCCACTACGAAGGCCCGTGAAGCTGCTGCTAAACGAGTCCGAGAGAAACGTAAACAGACTCCTAAGTTCATCTACGGACGTAAGGAGTAACTAAAGGTTACATAAGGAGGCCCTAAATGGATAAGTACGATAAGAACGTACCAAGTGATTATGATGGTCTGTTCCAAAAGGCTGCTGATGCCAACGGGGTCTCTTATGACCTTTTACGt                                                                                                                                                                                               <  1:225964/298‑1 (MQ=255)`  
                                                                                                                  `aaGGTCTGGAGTGAGAACCAGAAGAAACTCGAAGAGAAAGCTCGTGAGAAGGCTCTGGCTGATGTGAACAAGCGAGCACCTATAGTTGCCGCTACGAAGGCCCGTGAAGCTGCTGCTAAACGAGTCCAAGAGAAACGTAAACAGACTCCTAAGTTCATCTACGGACGTAAGGAGTAACTAAAGGCTACATAAGGAGGCCCTAAATGGATAAGTACGATAAGAACGTACCAAGTGATTATGATGGTCTGTTCCAAAAGGCTGCTGATGCCAACGGGGTCTCTTATGACCTTTTACGTAAAGt                                                                                                                                                                                          <  1:158555/301‑1 (MQ=255)`  
                                                                                                                    `ggTCTGGAGTGAGAACCAGAAGAAACTCGAAGAGAAAGCTCGTGAGAAGGCTCTGGCTTATGTGAACAAGCGAGCACCTATAGTTGCCGCTACGAAGGCCCGTGAAGCTGCTGCTAAACGAGTCCGAGAGAAACGTAAACAGACTCCTAAGTTCATCTACGGACGTAAGGAGTAACTAAAGGTTACATAAGGAGGCCCTAAATGGATAAGTACGATAAGAACGTACCAAGTGATTATGATGGTCTGTTCCAAAAGGCTGCTGATGCCAACGGGGTCTCTTATGACCTTTTACGTCAAGTcc                                                                                                                                                                                        >  1:26498/1‑300 (MQ=255)`  
                                                                                                                         `ggAGTGAGAACCAGAAGAAACTCGAAGAGAAAGCTCGTGAGAAGGCTCTGGCTGATGTGAACAAGCGAGCACCTATAGTTGCCGCTACGAAGGCCCGTGAAGCTGCTGCTAAACGAGTCCGAGAGAAACGTAAACAGACTCCTAAGTTCATCTACGGACGTAAGGAGTAACTAAAGGCTACATAAGGAGGCCCTAAATGGATAAGTACGATAAGAACGTACCAAGTGATTATGATGGTCTGTTCCAAAAGGCTGCTGATGCCAACGGGGTCTCTTATGACCTTTTACGTAAAGTCGCTTgg                                                                                                                                                                                   >  1:75937/1‑301 (MQ=255)`  
                                                                                                                           `ggTGAGAACCAGAAGAAACTCGAAGAGAAAGCTCGTGAGAAGGCTCTGGCTGATGGGAACAAGCGAGCACCTATAGTTGCCGCTACGAAGGCCCGTGAAGCTGCTGCTAAACGAGTCCGAGAGAAACGTAAACAGACTCCTAAGTTCATCTACGGACGTAAGGAGTAACTAAAGGCTACATAAGGAGGCCCTAAATGGATAAGTACGATAAGAACGTACCAAGTGATTATGATGGTCTGTTCCAAAAGGCTGCTGATGCCAACGGGGTCTCTTATGACCTTTTACGTAAAGTCGCTTGGAc                                                                                                                                                                                 <  1:313774/300‑1 (MQ=255)`  
                                                                                                                                 `aaCCAGAAGAAACTCGAAGAGAAAGCTCGTGAGAAGGCTCTGGCTGATGTGAACAAGCGAGCACCTATAGTTGCCGCTACGAAGGCCCGTGAAGCTGCTGCTAAACGAGTCCGAGAGAAACGTAAACAGACTCCTAAGTTCATCTACGGACGTAAGGAGTAACTAAAGGTTACATAAGGAGGCCCTAAATGGATAAGTACGATAAGAACGTACCAAGTGATTATGATGGTCTGTTCCAAAAGGCTGCTGATGCCAACGGGGTCTCTTATGACCTTTTACGTAAATTCGCTTGGACAGAAta                                                                                                                                                                           >  1:240830/1‑300 (MQ=255)`  
                                                                                                                                 `aaCCAGAAGAAACTCGAAGAGAAAGCTCGTGAGAAGGCTCTGGCTGATGTGAACAAGCGAGCACCTATAGTTGCCGCTACGAAGGCCCGTGAAGCTGCTGCTAAACGAGTCCGAGAGAAACGTAAACAGACTCCTAAGTTCATCTACGGACGTAAGGAGTAACTAAAGGCTACATAAGGAGGCCCTAAATGGATAAGTACGATAAGAACGTACCAAGTGATTATGATGGTCTGTTCCAAAAGGCTGCTGATGCCAACGGGGTCTCTTATGACCTTTTACGTAAAGTCGCTTGGACAGAATc                                                                                                                                                                           >  1:59163/1‑301 (MQ=255)`  
                                                                                                                                             `cgcGAAGAGAAAGCGCGTGAGAAGGCTCTGGCTGATGTGAACAAGCGAGCACCTATAGTTGCCGCTACGAAGGCCCGTGAAGCTGCTGCTAAACGAGTCCGAGAGAAACGTAAACAGACTCCTAAGTTCATCTACGGACGTAAGGAGTAACTAAAGGCTACATAAGGAGGCCCTAAATGGATAAGTACGATAAGAACGTACCAAGTGATTATGATGGTCTGTTCCAAAAGGCTGCTGATGCCAACGGGGTCTCTTATGACCTTTTACGTAAAGTCGCTTGGACAGAATCACGATTTGTGcc                                                                                                                                                               <  1:292697/299‑1 (MQ=255)`  
                                                                                                                                             `cTCGAAGAGAAAGCTCGTGAGAAGGCTCTGGCTGATGTGAACAAGCGAGCACCTATAGTTGCCGCTACGAAGGCCCGTGAAGCTGCTGCTAAACGAGTCCGAGAGAAACGTAAACAGACTCCTAAGTTCATCTACGGCCGTAAGGAGTAACTAAAGGTTACATAAGGAGGCCCTAAATGGATAAGTACGATAAGAACGTACCAAGTGATTATGATGGTCTGTTCCAAAAGGCTGCTGATGCCAACGGGGTCTCTTTTGACCTTTTACGTAAAGTCGCTTGGACAGAATCACAATTTGTGcc                                                                                                                                                               >  1:143201/1‑301 (MQ=255)`  
                                                                                                                                             `cTCGAAGAGAAAGCTCGTGAGAAGGCTCTGGCTGATGTGAACAAGCGAGCACCTATAGTTGCCGCTACGAAGGCCCGTGAAGCTGCTGCTAAACGAGTCCGAGAGAAACGTAAACAGACTCCTAAGTTCATCTACGGACGTAAGGAGTAACTAAAGGCTACATAAGGAGGCCCTAAATGGATAAGTACGATAAGAACGTACCAAGTGATTATGATGGTCTGTTCCAAAAGGCTGCTGATGCCAACGGGGTCTCTTATGACCTTTTACGTAAAGTCGCTTGGACAGAATCACGATTTGTGcc                                                                                                                                                               <  1:15300/301‑1 (MQ=255)`  
                                                                                                                                                            `cGTGAGAAGGCTCTGGCTGATGTGAACAAGCGAGCACCTATAGTTGCCGCTACGAAGGCCCGTGAAGCTGCTACTAAACGAGTCCGAGAGAAACGTAAACAGACTCCTAAGTTCATCTACGGACGTAAGGAGTAACTAAAGGTTACATAAGGAGGCCCTAAATGGATAAGTACGATAAGAACGTACCAAGTGATTATGATGGTCTGTTCCAAAAGGCTGCTGATGCCAACGGGGTCTCTTATTACCTTTTACGTAAAGTCGCTTGGACAGAATCACGATTTGTGCCTACAGCAAAATCTaa                                                                                                                                                >  1:172807/1‑301 (MQ=255)`  
                                                                                                                                                               `gagaAGGCTCTGGCTGATGTGAACAAGCGGGCACCTATAGTTGCCGCTACGAAGGCCCGTGAAGCGGCTGCTAAACGAGTCCGAGAGAAACGTAAACAGACTCCTAAGTTCATCTACGGACGTAAGGAGTAACTAAAGGCTACATAAGGAGGCCCTAAATGGATAAGTACGATAAGAACGTACCAAGTGATTATGATGGTCTGTTCCAAAAGGCTGCTGATGCCAACGGGGTCTCTTATGACCTTTTACGTAAAGTCGCTTGGACAGAATCACGATTTGTGCCTACAGCAAAATCTAAGAc                                                                                                                                             <  1:202480/301‑1 (MQ=255)`  
                                                                                                                                                                    `aaagagacaggATGTGAACAAGCGAGCACCTATAGTTGCCGCTACGAAGGCCCGTGAAGCTGCTGCTAAACGAGTCCGAGAGAAACGTAAACAGACTCCTAAGTTCATCTAAGGACGTAAGGAGTAACTAAAGGTTACATAAGGAGGCCCTAAATGGATAAGTACGATAAGAACGTACCAAGTGATTATGATGGTCTGTTCCAAAAGGCTGCTGATGCCAACGGGGTCTCTTATGACCTTTTACGTAAAGTCGCTTGGACAGAATCACGATTTGTGCCTACAGCAAAATCTAAGACTGGAc                                                                                                                                        <  1:320629/291‑1 (MQ=255)`  
                                                                                                                                                                            `gtGATGTGAACAAGCGAGCACCTATAGTTGCCGCTACGAAGGCCCGTGAAGCTGCTGCTAAACGAGTCCGAGAGAAACGTAAACAGACTCCTAAGTTCATCTACGGACGTAAGGAGTAACTAAAGGCTACATAAGGAGGCCCTAAATGGATAAGTACGATAAGAACGTACCAAGTGATTATGATGGTCTGTTCCAAAAGGCTGCTGATGCCAACGGGGTCTATTATGACCTTTTACGTAAAGTCGCTTGGACAGAATCACGATTTGTGCCTACAGCAAAATCTAAGACTGGACCATTAGGc                                                                                                                                <  1:16730/300‑1 (MQ=255)`  
                                                                                                                                                                            `gatgtgaaaaagagacagaagctagatgtGCCGATATGAAGGCGAGAGAAGATGATGAGAAAGGAGTACGAAAGAAACGTAAACAGACGCATAAGGTCAGCTACGGAAGGAAGGAGTAAGGAAAGGTGACAGAAGGAGGTCCTAGATGGATAAGTACGAGAAGAACGTACCAAGTGATTATGATGGTCTGTTCCAAAAGGATGCTGATGCGAACGGGGTCTCTTATGACCTTATACGGAAAGTCGCTTGGACAGAGTCACGATTTGTGCCTACAGCAAAATCTAAGACTGGACCATTAGGc                                                                                                                                <  1:328731/273‑1 (MQ=255)`  
                                                                                                                                                                             `tGATGTGAACAAGCGAGCACCTATAGTTGCCGCTACGAAGGCCCGTGAAGCTGCTGCTAAACGAGTCCGAGAGAAACGTAAACAGACTCCTAAGTTCATCTACGGACGTAAGGAGTAACTAAAGGTTACATAAGGAGGCCCTAAATGGATAAGTACGATAAGAACGTACCAAGTGATTATGATGGTCTGTTCCAAAAGGCTGCTGATGCCAACGGGGTCTCTTATGACATTTTACGTAAAGTCGCTTGGACAGAATCACGATTTGTGCCTACAGCAAAATCTAAGACTGGACCATTAGGCa                                                                                                                               <  1:170694/301‑1 (MQ=255)`  
                                                                                                                                                                                 `gtgAACAAGCGAGCACCTATAGTTGCCGCTACGAAGGCCCGTGAAGCTGCTGCTAAACGAGTCCTAGAGAAACGTAAACAGACTCCTAAGTTCATCTACGGACGTAAGGAGTAACTAAAGGTTACATAAGGAGGCCCTAAATGGATAAGTACGATAAGAACGTACCAAGTGATTATGATGGTCTGTTCCAAAAGGCTGCTGATGCCAACGGGGTCTCTTATGACCTTTTACGTAAAGTCGCTTGGACAGAATCACGATTTGTGCCTACAGCAAAATCTAAGACTGGACCATTAGGCatgat                                                                                                                           >  1:86504/1‑301 (MQ=255)`  
                                                                                                                                                                                        `aGCGAGCACCTATAGTTGCCGCTACGAAGGCCCGTGAAGCTGCTGCTAAACGAGTCCGAGAGAACCGTAAACAGACTCCTAAGTTCATCTACGGACGTAAGGAGTAACTAAAGGCTACATAAGGAGGCCCTAAATGGATAAGTACGATAAGAACGTACCAAGTGATTATGATGGTCTGTTTTAAAAGGCTGCTGCTGCCATCGGGGTCTCTTATGACCTTTTACGTAAACTCGCTTGGACAGAATCACGATTTGTGCCTACAGCAAAATCTAAGACTGGACCATTAgccctgtctcttata                                                                                                                    >  1:331851/1‑287 (MQ=255)`  
                                                                                                                                                                                                `ccTATAGTTGCCGCTACGAAGGCCCGTGAAGCTGCTGCTAAACGAGTCCGAGAGAAACGTAAACAGACTCCTAAGTTCATCTACGGACGTAAGGAGTAACTAAAGGCTACATAAGGAGGCCCTAAATGGATAAGTACGATAAGAACGTACCAAGTGATTATGATGGTCTGTTCCAAAAGGCTGCTGATGCCAACGGGGTCTCTTATGACCTTTTACGTAAAGTCGCTTGGACAGAATCACGATTTGTGCCTACAGCAAAATCTAAGACTGGACCATTAGGCATGATGCAATTTACCAAGGc                                                                                                            <  1:111879/301‑1 (MQ=255)`  
                                                                                                                                                                                                             `cTACGAAGGCCCGTGAAGCTGCTGCTAAACGAGTCCGAGAGAAACGTAAACAGACTCCTAAGTTCATCTACGGACGTAAGGAGTAACTAAAGGCTACATAAGGAGGCCCTAAATGGATAAGTACGATAAGAACGTACCAAGTGATTATGATGGTCTGTTCCAAAAGGCTGCTGATGCCAACGGGGTCTCTTATGACCTTTTACGTAAAGTCGCTTGGACAGAATCACGATTTGTGCCTACAGCAAAATCTAAGACTGGACCATTAGGCATGATGCAATTTACCAAGGCAACCGcctgtctc                                                                                               >  1:66817/1‑294 (MQ=255)`  
                                                                                                                                                                                                                  `ttgtataagatacaggctgctAAACGAGTCCGAGAGAAACGTAAACAGACTCCTAAGTTCATCTACGGACGTAAGGAGTAACTAAAGGTTACATAAGGAGGCACTAAATGGATAAGTACGATAAGAACGTACCAAGTGATTATGATGGTCTGTTCCAAAAGGCTGCTGATGCCAACGGGGTCTCTTATGACCTTTTACGTAAAGTCGCTTGGACAGAATCACGATTTGTGCCTACAGCAAAATCTAAGACTGGACCATTAGGCATGATGCAATTTACCAAGGCAACCGCTAAGGCCCTCgg                                                                                          <  1:272431/286‑1 (MQ=255)`  
                                                                                                                                                                                                                       `ccGTTAAGCTGCTGCTAAACGAGTCCGAGAGAAACGTAAACAGACTCCTAAGTTCATCTACGGACGTAAGGAGTAACTAAAGGCTACATAAGGAGGCCCTAAATGGATAAGTACGATAAGAACGTACCAAGTGATTATGATGGTCTGTTCCAAAAGGCTGCTGATGCCAACGGGGTCTCTTATGACCTTTTACGTAAAGTCGCTTGGACAGAATCACGATTTGTGCCTACAGCAAAATCTAAGACTGGACCATTAGGCATGATGCAATTTACCAAGGCAACCGCTAAGGCCCTCGGTCTGc                                                                                     >  1:252260/1‑301 (MQ=255)`  
                                                                                                                                                                                                                       `ccGTGAAGCTGCTGCTAAACGAGTCCGAGAGAAACGTAAACAGACTCCTAAGTTCATCTACGGACGTAAGGAGTAACTAAAGGTTACATAAGGAGGCCCTAAATGGATAAGTACGATAAGAACGTACCAAGTGATTATGATGGTCTGTTCCAAAAGGCTGCTGATGCAAACGGGGTCTCTTATGACCTTTTACGTAAAGTCGCTTGGACAGAATCACGATTTGTGCCTACAGCAAAATCTAAGACTGGACCATTAGGCATGATGCAATTTACCAAGGCAACCGCTAAGGCCCTCGGttcgc                                                                                     >  1:207367/1‑297 (MQ=255)`  
                                                                                                                                                                                                                          `tGAAGCTGCTGCTAAACGAGTCCGAGAGAAACGTAAACAGACTCCTAAGTTCATCTACGGACGTAAGGAGTAACTAAAGGCTACATAAGGAGGCCCTAAATGGATAAGTACGATAAGAACGTACCAAGTGATTATGATGGTCTGTTCCAAAAGGCTGCTGATGCCAACGGGGTCTCTTATGACCTTTTACGTAAAGTCGCTTGAACAGAATCACGATTTGTGCCTACAGCAAAATCTAAGACTGGACCATTAGGCATGATGCAATTTACCAAGGCAACCGCTAAGGCCCTCGGTCTGCGAg                                                                                  <  1:47320/301‑1 (MQ=255)`  
                                                                                                                                                                                                                                            `ataagagacagaaCGTAAACAGACTCCTAAGTTCATCTACGGACGTAAGGAGTAACTAAAGGCTACATAATGAGGCCCTAAATGGATAAGTACGATAAGAACGTACCAAGTGATTATGATGGTCTGTTCCAAAAGGCTGCTGATGCCAACGGGGTCTCTTATGACCTTTTACGTAAAGTCGCTTGGACATAATCACGATTTGTGCCTACAGCAAAATCTAAGACTGGACCATTAGGCATGATGCAATTTACCAAGGCAACCGCTAAGGCCCTCGGTCTGCGAGTTACCGATGGTCCAgacg                                                                <  1:124028/290‑1 (MQ=255)`  
                                                                                                                                                                                                                                                      `aaaCGTAAACAGACTCCTAAGTTCATCTACGGACGTAAGGAGTAACGAAAGGTTACATAAGGAGGCCCTAATTGGATAAGTACGATAAGAACGTACCAAGTGATTATGATGGTCTGTTCCAAAAGGCTGCTGATGCCAACGGGGTCTCTTATGACCTTTTACGTAAAGTCGCTTGGACAGAATCACGATTTGTGCCTACAGCAAAATCTAAGACTGGACCATTAGGCATGATGCAATTTACCAAGGCAACCGCTAAGGCCCTCGGTCTGCGAGTTACCGATGGTCCAGACGACGACCGACt                                                      <  1:342352/301‑1 (MQ=255)`  
                                                                                                                                                                                                                                                       `aaCGTAAACAGACTACTAAGTTCATCTACGGACGTAAGGAGTAACTAAAGGTTACATAAGGAGGCCCTAAATGGATAAGTACGATAAGAACGTACAAAGTGATTATGATGGTCTGTTCCAAAAGGCTGCTGATGCCAACGGGGTCTCTTATGACCTTTTACGTAAAGTCGCTTGGACAGAATCACGATTTGTGCCTACAGCAAAATCTAAGACTGGACCATTAGGCATGATGCAATTTACCAAGGCAACCGCTAAGGCCCTCGGTCTGCGAGTTACCAATGGTCCAGACGACGACCGACTg                                                     >  1:64090/1‑301 (MQ=255)`  
                                                                                                                                                                                                                                                          `gTAAACAGACTCCTAAGTTCATCTACGGACGTAAGGAGTAACTAAAGGCTACATAAGGAGGCCCTAAATGGATAAGTACGATAATAACGTACGAAGTGATTATGATGGTCTGTTCCAAAAGGCTGCTTATGCCAACGGGGTCTCTTATGACCTTTTACGTAAAGTCGCTTGGACAGAATCACGATTTGTGCCTACAGCAAAATCTAAGACTGGACCATTGGGCATGATGCAATTTACCAAGGCAACCGCTAAGGCCCTCGGTCTGCGAGTTACCGATGGTCCAGACGACGACCGACTGAAc                                                  <  1:309314/301‑1 (MQ=255)`  
                                                                                                                                                                                                                                                            `aatcAGACTCCTAAGTTCATCTACGGACGTAAGGAGTAACTAAAGGTTACATAAGGAGGCCCTAAATGGATAAGTACGATAAGAACGTACCAAGTGATTATGATGGTCTGTTCCAAAAGGCTGCTGATGCCAACGGGGTCTCTTATGACCTTTTACGTAAAGTCGCTTGGACAGAATCACGATTTGTGCCTACAGCAAAATCTAAGACTGGACCATTAGGCATGATGCAATTTACCAAGGCAACCGCTAAGGCCCTCGGTCTGCGAGTTACCGATGGTCCAGACGACGACCGACTGAAccc                                                <  1:115528/298‑1 (MQ=255)`  
                                                                                                                                                                                                                                                              `gggacagccTAAGTTCATCTACGGACGTAAGGAGTAACTAAAGGTTACATAAGGAGGCCCTAAATGGATAAGTACGATAAGAACGTACCAAGTGATTATGATGGTCTGTTCCAAAAGGCTGCTGATGCCAACGGGGTCTCTTATGACCTTTTACGTAAAGTCGCTTGGACAGAATCACGATTTGTGCCTACAGCAAAATCTAAGACTGGACCATTAGGCATGATGCAATTTACCAAGGCAACCGCTAAGGCCCTCGGTCTGCGAGTTACCGATGGTCCAGACGACGACCGACTGAACCCTg                                              <  1:287992/294‑1 (MQ=255)`  
                                                                                                                                                                                                                                                               `cAGACTCCTAAGTTCATCTACGGACGTAAGGAGTAACTAAAGGTTACATAAGGAGGCCCTAAATGGATAAGTACGATAAGAACGTACCAAGTGATTATGATGGTCTGTTCCAAAAGGCTGCTGATGCCAACGGGGTCTCTTATGACCTTTTACGTAAAGTGGCTTGGACAGAATAACGATTGGTGCTTACAGCAAAATCTAAGACTGAACAATAAGGCATGAGGCAATTTACCAAGGCAACCGCTAAGGCCCTCGGTCTGCGGGTTACGGATGGTCAAGACGACGACCGACGGAAccccgg                                             >  1:198902/1‑298 (MQ=255)`  
                                                                                                                                                                                                                                                                   `cTCCTAAGTTCATCTACGGACGTAAGGAGTAACTAAAGGCTACATAAGGAGGCCCTAAATGGATAAGTACGATAAGAACGTACCAAGTGATTATGATGGTCTGTTCCAAAAGGCTGATGATGCCAACGGGGTCTCTTATGACCTTTTACGTAAAGTCGCTTGGACAGAATCACGATTTGTGCCTACAGCAAAATCTAAGACTGGACCATTAGGCATGATGCAATTTACCAAGGCAACCGCTAAGGCCCTAGGTCTGCGAGTTACCGATGGTCCAGACGACGACCGACTGAAcccctgtctc                                         >  1:323809/1‑294 (MQ=255)`  
                                                                                                                                                                                                                                                                   `cTCCTAAGTACATCTACGGACGTAAGGAGTAACTAAAGGTTACATAAGGAGGCCCTAAATGGATAAGTACGATAAGAACGTACCAAGTGATTATGATGGTCTGTTCCAAAAGGCTGCTGATGCCAACGGGGTCTCTTATGACCTTTTACGTAAAGTCGCTTGGACAGAATCACGATTTGTGCCTACAGCAAAATCTAAGACTGGACAATTAGGAATGATGCAATTTACCAAGGCAACCGCTAAGGCCCTCGGTCTGCGAGTTACCGATGGTCCAGACGACGACCGACTGAACCCTGAGTTa                                         >  1:284847/1‑301 (MQ=255)`  
                                                                                                                                                                                                                                                                      `aagagaaagtCTACGTAAGGAAGGAGTAACTAAAGGGTAAATAAGGAGGCCCTAAATGGATAAGTACGAAAAGAAAGTACCAAGTGATTATGATGGTCTGGTCCAAAAGGCTGCTGATGCCAACGGGGTCTCTTATGACCTTTTACGTAAAGTCGCGTGGACAGAATCAAGATTTGTGCCTACAGCAAAATCTAAGACTGGACCATTAGGCATGATGCAATTTACCAAGGCAACCGCTAAGGCCCTCGGTCTGCGAGTTACCGATGGTCCAGACGACGACCGACTGAACCCTGAGTTAGCt                                      <  1:13960/292‑1 (MQ=255)`  
                                                                                                                                                                                                                                                                        `aaagagacagaCGGACGTAAGGAGTAACTAAAGGCTACATAAGGATGCCCTAAATGGATAAGTAAGATAAGAACGTACCAAGTGATTATGATGGTCTGATCCAAAAGGCTGCTGATGCCAACGGGGTCTCTTATGACCTTTTACGTAAAGTCGCTTGGACAGAATCACGATTTGTACCTACAGCAAAATCTAAGACTGGACCATTAGGCATGATGCAAGTTACCAAGGCAACCGCTAAGGCCCTCGGTCTGCGAGTTACCGATGGTCCAGACGACGACCGACTGAACCCTGAGTTAGCTAt                                    <  1:244376/291‑1 (MQ=255)`  
                                                                                                                                                                                                                                                                         `aGTTCATCTACGGACGTAAGGAGTAACTAAAGGTTACATAAGGAGGCCCTAAATGGATAAGTACGATAAGAACGTACCAAGTGATTATGATGGTCTGTTCCAAAAGGCTGCTGATGCCAACGGGGTCTCTTATGACCTTTTACGTAAAGTCGCTTGGACAGAATCACGATTTGTGCCTACAGCAAAATCTAAGACTGGACCATTAGGCATGATGCAATTTACCAAGGCAACCGCTAAGGCCCTCGGTCTGCGAGTTACCGATGGTCCAGACGACGACCGACTGAACCCTGAGTTAGCTAtt                                   <  1:161166/301‑1 (MQ=255)`  
                                                                                                                                                                                                                                                                             `cATCTACGGACGTAAGGAGTAACTAAAGGTTACATAAGGAGGCCCTAAATGGATAAGTACGATAAGAACGTACCAAGTGATTATGATGGTCTGTTCCAAAAGGCTGCTGATGCCAACGGGGTCTCTTATGACCTTTTACGTAAAGTCGCTTGGACAGAATCACGATTTGTGCCTACAGCAAAATCTAAGACTGGACCATTAGGCATGATGCAATTTACCAAGGCAACCGCTAAGGCCCTCGGTCTGCGAGTTACCGATGGTCCAGACGACGACCGACTGAACCCTGAGTTAGCTATTAAtg                               <  1:89110/301‑1 (MQ=255)`  
                                                                                                                                                                                                                                                                                `cTACGGACGTAAGGAGTAACTAAAGGTTACATAAGGAGGCCCTAAATGGATAAGTACGATAAGAACGTACCAAGTGATTATGATGGTCTGTTCCAAAAGGCTGCTGATGCCAACGTGGTCTCTTATGACCTTTTACGTAAAGTCGCTTGGACAGAATCACGATTTGTGCCTACAGCAAAATCTAAGACTGGACCATTAGGCATGATGCAATTTACCAAGGCAACCGCTAAGGCCCTCGGTCTGCGAGTTACCGATGGTCCAGACGACGACCGCCTGAACCCTGAGTTAGCTATTAAtgctg                            >  1:127908/1‑301 (MQ=255)`  
                                                                                                                                                                                                                                                                                     `gACGTAAGGAGTAACTAAAGGTTACATAAGGAGGCCCTAAATGGATAAGTACGATAAGAACGTACCAAGTGATTATGATGGTCTGTTCCAAAAGGCTGCTGATGCCAACGGGGTCTCTTATGACCTTTTACGTAAAGTCGCTTGGACAGAATCACGATTTGTGCCTACAGCAAAATCTAAGACTGGACCATTAGGCATGATGCAATTTACCAAGGCAACCGATAAGGCCCTCGGTCTGCGAGTTACCGATGGTCCAGACGACGACCGACTGAACCCTGAGTTAGCTATTAATGCTGCCGCt                       >  1:192630/1‑301 (MQ=255)`  
                                                                                                                                                                                                                                                                                     `gACGTAAGGAGTAACTAAAGGCTACATAAGGAGGCCCTAAATGGATAAGTACGATAAGAACGTACCAAGTGATTATGATGGTCTGTTCCAAAAGGCTGCTGATGCCAACGGGGTCTCTTATGACCTTTTACGTAAAGTCGCTTGGACAGAATCACGATTTGTGCCTACAGCAAAATCTAAGACTGGACCATTAGGCATGATGCAATTTACCAAGGCAACCGCTAAGGCCCTCGGTCTGCGAGTTACCGATGGTCCAGACGACGACCGACTGAACCCTGAGTTAGCTATTAATGCTGCCGCt                       >  1:148341/1‑301 (MQ=255)`  
                                                                                                                                                                                                                                                                                     `gACGTAAGGAGTAACTAAAGGCTACATAAGGAGGCCCGAAATGGATAAGTACGATAAGAACGTACCAAGTGATTATGAGGGTCTGTTCCAAAAGGCTGCTGATGCAAACGGGGACTCTTATGACCTTTTACGTAAAGTCGCTAGGACAGAATCAGGATTTGTGCCGACAGAAAAAGCTAAGACTGGACCAGTAAGCAAGATGAAATTTACCAAGGCAACCGCAAAGGCACTCGGTATGAGAGTAACCGAAGGTACAGACGACGACCGAATGAACACGGAGtctggccgtgatacagatcgc                       >  1:326219/1‑281 (MQ=255)`  
                                                                                                                                                                                                                                                                                       `cgaaaGCAGTAACTAAAGGCTACATAAGGAGGTCCTAAATGGATAAGAACGATAAGAACGTACCAAGTGATTATGATGGTCTGTATCAAAAGGCTGCTGATGCCAACGGGGTCTCTTATGACCTTTTACGTAAAGTCGCTTGGACAGAATCACGATTTGTGCCTACAGCAAAATCTAAGACTGGACCATTAGGCATGATGCAATTTACCAAGGCAACCGCTAAGGCCCTCGGTCTGCGAGTTACCGATGGTCCAGACGACGACCGACTGAACCCTGAGTTAGCTATTAATGCTGCCACTaa                     <  1:296299/298‑1 (MQ=255)`  
                                                                                                                                                                                                                                                                                             `gAGTAACTAAAGGCTACATAAGGAGGCCCTAAATGGATAAGTACGATAAGAACGTACCAAGTGATTATGATGGTCTGTTCCAAAAGGCTGCTGATGCCAACGGGGTCTCTTATGACCTTTTACGTAAAGTCGCTTGGACAGAATCACGATTTGTGCCTACAGCAAAATCTAAGACTGGACCATTAGGCATGATGCAATTTACCAAGGCAACCGCTAAGGCCCTCGGTCTGCGAGTTACCGATGGTCCAGACGACGACCGACTGAACCCTGAGTTAGCTATTAATGCTGCCGCtgtctctta               >  1:184418/1‑293 (MQ=255)`  
                                                                                                                                                                                                                                                                                                   `cTAAAGGCTACATAAGGAGGCCCTAAATGGATAAGTACGATAAGAACGTACCAAGTGATTATGATGGTCTGTTCCAAAAGGCTGCTGATGCCAACGGGGTCTCTTATGACCTTTTACGTAAAGTCGCTTGGACAGAATCACGATTTGTGCCTACAGCAAAATCTAAGACTGGACCATTAGGCATGATGCAATTTACCAAGGCAACCGCTAAGGCCCTCGGTCTGCGAGTTACCGATGGTCCAGACGACGACCGACTGAACCCTGAGTTAGCTATTAATGCTGCCGCTAAGCAACTTGCAgg         <  1:107763/301‑1 (MQ=255)`  
                                                                                                                                                                                                                                                                                                       `aGGTTACATAAGGCGGCCCTAAATGGATAAGTACGATAAGAACGTACCAAGTGATTATGATGGTCTGTTCCAAAAGGCTGCTGATGCCAACGGGGTCTCTTATGACCTTTTACGTAAAGTCGCTTGGACAGAATCACGATTTGTGCCTACCGCAAAATCTAAGACTGGACCATTAGGCGTGATGCAATTTACCAAGGCAACCGCTCAGGCCCTCGGTCTGCGAGTTACCGCTGGTCCAGACGACGACCGACTGAACCCTGAGTTAGCTATTAATGCTGCCTCTAAGCACCTTGCATGTCTg     >  1:237482/1‑301 (MQ=255)`  

                                                                                                                                                                                                                                                                                                          `|`                                                                                                                                                                                                                                                                                                              
`AGCAACCCTTGGATAACCAATAAGCAACTGACCATGTATTCTCAAGGTGACTCCATTTACCTTATGGACACCACAGGTCAAGTCAGAGTCCGATACGACAAAGAGTTACTCTCGAAGGTCTGGAGTGAGAACCAGAAGAAACTCGAAGAGAAAGCTCGTGAGAAGGCTCTGGCTGATGTGAACAAGCGAGCACCTATAGTTGCCGCTACGAAGGCCCGTGAAGCTGCTGCTAAACGAGTCCGAGAGAAACGTAAACAGACTCCTAAGTTCATCTACGGACGTAAGGAGTAACTAAAGGCTACATAAGGAGGCCCTAAATGGATAAGTACGATAAGAACGTACCAAGTGATTATGATGGTCTGTTCCAAAAGGCTGCTGATGCCAACGGGGTCTCTTATGACCTTTTACGTAAAGTCGCTTGGACAGAATCACGATTTGTGCCTACAGCAAAATCTAAGACTGGACCATTAGGCATGATGCAATTTACCAAGGCAACCGCTAAGGCCCTCGGTCTGCGAGTTACCGATGGTCCAGACGACGACCGACTGAACCCTGAGTTAGCTATTAATGCTGCCGCTAAGCAACTTGCAGGTCTGGTA  >  NC_001604.1/30278‑30876`

  

Alignment Legend

Aligned base mismatch/match (shaded by quality score): `ATCG/ATCG` `< 3 ≤` `ATCG/ATCG` `< 11 ≤` `ATCG/ATCG` `< 22 ≤` `ATCG/ATCG` `< 34 ≤` `ATCG/ATCG` `< 38 ≤` `ATCG/ATCG`

Unaligned base: `atcg`    Masked matching base: `atcg`    Alignment gap: `‑`    Deleted base: `‑`
